# Contamination Level, Distribution, and Inventory of Dechlorane Plus (DP) in the Surface Soil of Shenyang City, China

**DOI:** 10.3390/toxics13050335

**Published:** 2025-04-24

**Authors:** Hui Wang, Siyi Yu, Tony R. Walker, Hao Wu, Xiaoxu Wang, Yueli Yang, Yinggang Wang

**Affiliations:** 1 Key Laboratory of Ecological Restoration of Regional Contaminated Environment, Ministry of Education, College of Environment, Shenyang University, Shenyang 110044, China; 15114191298@163.com (S.Y.); zhu13mao@163.com (H.W.); 13840554151@163.com (X.W.); 2 Northeast Geological S&T Innovation Center of China Geological Survey, Shenyang 110000, China; 3 School for Resource and Environmental Studies, Dalhousie University, Halifax, NS B3H 4R2, Canada; trwalker@dal.ca; 4 Department of Ecology and Environment of Liaoning Province, Shenyang 110161, China

**Keywords:** Dechlorane Plus (DP), surface soil, Shenyang city, DP isomers

## Abstract

Dechlorane Plus (DP), an emerging type of persistent organic pollutant (POP), poses potential harmful effects on plants, animals, and humans alike, garnering increasing attention. Urban surface soil is easily accessible to urban residents, and its environmental conditions have a more significant impact on urban residents. However, there are few studies on related DP contamination. In this study, the contamination of DP in surface soil from Shenyang City, Liaoning Province, China, was investigated. Soil samples were collected from 33 different locations in May and June 2023. The total DP (∑DP), anti-DP, and syn-DP were determined by gas chromatography and ranged from not detected (ND) to 77.80 ng/g, from ND to 61.50 ng/g, and from ND to 16.30 ng/g, respectively. The mean values were 33.60 ± 18.93 ng/g, 27.01 ± 14.32 ng/g, and 8.57 ± 4.55 ng/g. The findings indicate that anti-DP is more readily detectable than syn-DP, attributable to the lower proportion of syn-DP in the overall DP production and the distinct physicochemical properties of DP isomers. The fsyn [syn-DP/(anti-DP + syn-DP)] is 0.14–0.40, with a mean value of 0.22. This aligns closely with the values observed in commercial DP formulations, suggesting that the primary sources are derived from commercial DP products. Contour maps show that DP concentrations are influenced by urban land use and DP production. Based on the Tyson polygon method, the DP inventory was calculated at approximately 1.18 tons, with the unit area load exceeding previously reported values. The results also show that the health risks of DP are minimal, but children are more susceptible to the impacts of DP than adults, and oral ingestion is a more critical exposure pathway.

## 1. Introduction

Dechlorane Plus (DP), a halogenated flame retardant with high chlorine content, has been extensively incorporated into various industrial polymeric materials since it was introduced by Hooker Chemical (USA) in the 1960s [1,2,3]. Since it was detected in the North American Great Lakes Basin by Hoh et al. (2006) [4], DP has now been widely detected globally, in Korea [5], Pakistan [6,7], Vietnam [8], Canada [9], France [10], Japan [11], and China [12,13]. Furthermore, DP is highly persistent with a long half-life [14], and it is now omnipresent in multiple environmental media, for example, water bodies [15,16] and sediments [12,17,18]. DP has even been identified in multiple biological matrices, including edible fish, wild animals, even human bodies [13,19,20,21]. Thus, DP has become a global environmental concern. Once DP enters the environment, it demonstrates significant potential for absorption and biomagnification in both flora and fauna, leading to harmful effects on plants and animals [22,23,24,25]. DP enters the plant body and affects the photosynthesis of the plant body by affecting the enzyme, and affects the growth, reproduction, and normal metabolic process of the plant [22,26]. Animal toxicity studies also suggest that when DP reaches a particular mass concentration in animals, it will affect their metabolism, antioxidant system, reproductive ability, cell proliferation and apoptosis, damage their liver, brain, and genes, and even cause aberrations [25]. Upon entering the human body, DP could accumulate in biological tissues and organs, thereby posing potential health hazards.

DP is generally considered to come from production facilities, e-waste dismantling, and DP commercial products [7,27,28]. It has been reported that DP concentrations in urban and industrial zones are higher than in suburban areas due to the extensive use of DP commercial products [29,30]. Studies showing that DP concentrations in soil, sediment, atmosphere, aquatic organisms, and even human serum near electronic waste dismantling areas and DP production facilities were significantly higher than other areas, demonstrating that they may be important sources of DP pollution [31,32,33].

Although there have been some studies on DP in China and globally, it is still an emerging research area, especially since DP was listed in Annex A of the Stockholm Convention (Elimination Category). Research on DP in urban has become a recent focus of environmental research, especially since half the global population lives in cities [34], where it can accumulate in human tissues and organs, which affects nervous systems, child development, and causes diseases. Meanwhile, urban soil, especially surface soil, was easily affected by human activity, and it was an essential sink of contaminants [35,36]. The contaminants could deposit into the surface soil [37] and transfer to other environmental media, even enter into living organisms through ingestion, inhalation, dermal routes, etc. [35]. Therefore, as one of the most essential environmental mediums in cities, it is vital to study the contamination of DP in urban surface soil.

Shenyang, Northeast China’s largest urban center, hosts the extensive production of electrical wiring, cables, and polymeric materials incorporating DP, which are ubiquitously utilized in consumer goods and represent a significant environmental exposure pathway for this chlorinated flame retardant. Still, there have been few studies on DP pollution in the surface soil had been conducted [38,39]. This study aimed to fill a significant knowledge gap by measuring ∑DP, anti-DP, and syn-DP concentrations in surface soil in Shenyang City. The fractional abundances of DP isomers, spatial distribution, inventory, and the health risk of DP in soil were also studied. This study helps provide helpful information for future research directions to address this growing global pollutant.

## 2. Materials and Methods

### 2.1. Study Area and Sampling

This study was carried out within the second-ring road (the main urban area) of Shenyang City, which is the largest city in Northeast China. As the center of economy, politics, and culture in Northeast China, the area of Shenyang is 12,948 km^2^, and the population was estimated to be 9.20 million inhabitants in 2024. Meanwhile, Shenyang, which has attracted many heavy industrial factories since the 1950s, is famous as one of the most important heavy industry base and of the cradle of machine and equipment of manufacture industry China. By revitalizing the economy, the manufacturing enterprises were relocated outside the second-ring road of Shenyang City, and the land was re-zoned for development. To meet the needs of economic development and the daily lives of a large number of urban residents, a large amount of various industrial polymeric materials containing DP are being used, and this may pose a potential threat to the physical health of the residents.

**Figure 1:**
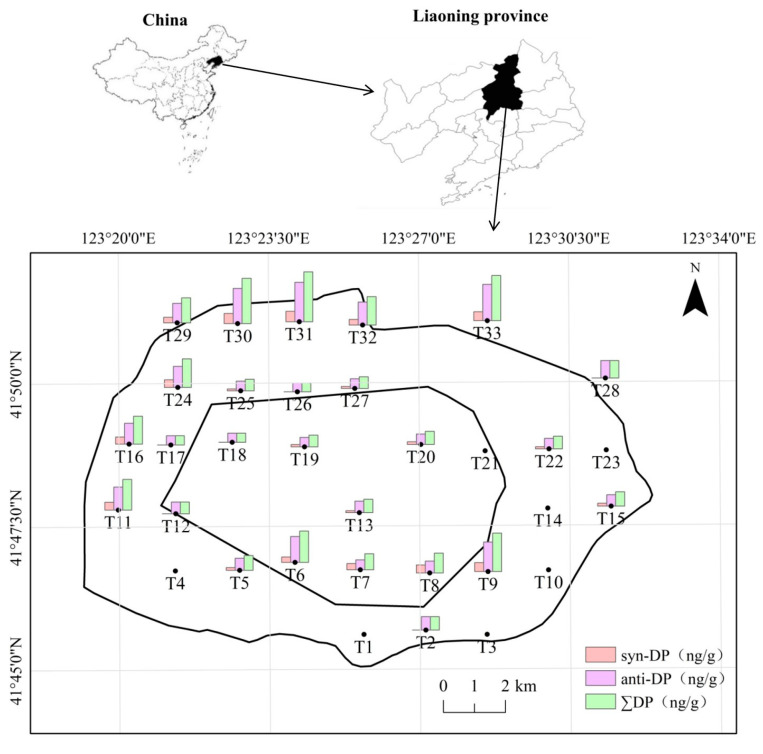
Map of sampling sites and DP isomer concentrations in surface soil in Shenyang, Northeast China.

Soil specimens were acquired from 33 spatially distributed sites within the second-ring road (the main urban area) of Shenyang City and were evenly distributed across the city (Figure 1). Sample sites were divided into four land-use areas, including residential (T1, T2, T4, T5, T8-T13, T18-T21, T23, T24, T27, T28, T31, and T33), business (T16 and T17), traffic (T3, T15, T29, and T30), and parkland (T6, T7, T14, T22, T25, T26, and T32). The surface soil samples (0–20 cm), which can easily to come into contact with people and have a more significant impact, were collected using five-point sampling in May and June of 2023. About 200 g soil were sampled at each site, and triplicate samples collected were used to determine mean concentrations and standard deviations (SDs). A stainless steel shovel was used to collect the soil samples. Soil samples wrapped with tinfoil were immediately transported to the laboratory and stored at −20 °C in a cryogenic refrigerator pending analysis.

### 2.2. Sample Preparation and Analysis

After the removal of coarse material, plant roots and other foreign matter, soil samples were air-dried to a constant weight, pulverized with an agata grinder, and sieved through a 150 μm mesh. DP was extracted and analyzed following previous methods [32,40], with some alterations. DP in urban soil samples was extracted using an accelerated solvent extractor (Dionex ASE300, Dionex, Sunnyvale, CA, USA), and acetone/hexane mixture (1:1) was used as extractant to the extraction of DP [32]. Then, the concentrated samples underwent a cleaning and fractionation process on acid–basic multilayer silica gel columns. A 20 mL mixture of dichloromethane and hexane in a 1:1 ratio was employed as the eluant [40]. The eluant was blown to nearly dry nitrogen flow, and then 1 mL hexane (HPLC grade) was added to redissolve the residue. The eluant was sealed and stored at <−20 °C until analyzed.

DP was determined using a gas chromatograph (Varian CP3800, Varian, Cary, NC, USA), and the chromatography analysis method of DP were established by 30 m × 0.32 mm × 0.25 μm CP-sil8CB quartz capillary chromatographic column. The flow capacity of highly purified nitrogen was 1 mL/min. The injection port temperature and detector temperature were held at 280 °C and 300 °C, respectively. The GC oven temperature program was as follows: held at 100 °C for 4 min, heated to 150 °C at 8 °C/min, held for 4 min, and then 12 °C/min to 300 °C, held for 13 min. An example gas chromatogram of the sample is shown in Figure S1.

### 2.3. Human Exposure and Health Risk Model

DP could enter into human bodies with urban surface soil by oral ingestion and dermal absorption, and the total daily exposure dose (*DED_t_*), oral ingestion (*DED_i_)*, and dermal absorption (*DED_da_*) could be calculated as followed in methods of previous studies [41,42]:

(1) $$DED_{i}=\frac{C\times DIR\times IEF}{BW}$$

(2) $$DED_{da}=\frac{C\times BSA\times SAS\times AF\times IEF}{BW\times 1000}$$

(3) $$DED_{t}=DED_{i}+DED_{da}$$

where *C* means the concentration of DP (ng/g) in urban surface dust; *DIR* means daily ingestion rate (g/d); *BW* is the body weight (kg); *BSA* is body surface area (cm^2^); *SAS* is soil adhered to skin (mg/(cm^2^ d)); *AF* is the fraction of DP absorbed in the skin, and *IEF* is the exposure fraction (hours spent over a day in an indoor environment). All values of the parameters for the five age groups can be found in Table S1.

Studies on the risk of DP were conducted. However, they are still in their infancy, with little available toxicological data, and previous studies show that DP has a non-carcinogenic risk [11,41,43]. In this study, the non-cancer health risk of DP was calculated as the hazard quotient (*HQ*) as following equations [42]:

(4) $$HQ=DED_{t}/RfD$$

where *RfD* means the reference dose for DP, and *RfDi* (oral ingestion reference doses) and *RfD_da_* (dermal absorption reference doses) were 5 and 2 mg kg-bw^−1^ d^−1^, respectively [44]. When *HQ* < 1, the health risk was acceptable.

### 2.4. Quality Assurance/Quality Control (QA/QC)

The contents of DP in the soil samples in this study were expressed based on dry weight (d.w.), and reagent blanks and duplicates were used every 12 soil samples to ensure the correct identification and quantification of target compounds during analysis. DP in reagent blanks was undetectable or <1% of mean DP concentrations in this study [3]. If the deviation between duplicate measurements was greater than 15%, the instruments were recalibrated. To ensure the accuracy of the analysis, the standard curve was examined daily using the DP reference standard purchased from AccuStandard (New Haven, CT, USA). The method detection limit (MDL) was determined as the analyte concentration yielding a signal-to-noise (S/N) ratio of 3:1 (peak-to-peak), and the MDL ranged from 10.1 to 14.3 pg/g (dry weight), reflecting matrix-dependent variations across soil samples. The recovery of spiked experiments was 90.01 ± 4.10% (mean ± SD). Solvents, including acetone, hexane, and dichloromethane, were purchased from Sinopharm Chemical Reagent Co., Ltd. (Shanghai, China).

### 2.5. Statistical Analysis

Statistical analysis was performed using Microsoft Excel (Microsoft, Redmond, WA, USA) and SPSS Statistics (version 22.0, SPSS, Chicago, IL, USA), and a map of sampling sites, concentrations of DP isomers, and contour map of DP were plotted by ArcGIS 10.5. Kriging interpolation was applied to the contour of DP concentrations (ng/g) in the soil.

## 3. Results and Discussion

### 3.1. DP Concentrations in Soil Samples

The mean, median, range, SD, and frequencies of detection (FDs) of DP isomers detected in surface soil are summarized in Table 1 and Figure 1. ∑DP, anti-DP, and syn-DP were detected in most of the 33 soil samples, and were 26, 26, and 20 for ∑DP, anti-DP, and syn-DP, respectively. Mean ∑DP concentrations were 33.60 ng/g, ranging from ND to 77.80 ng/g. Anti-DP concentrations ranged from ND to 61.50 ng/g with a mean of 27.01 ± 14.32 ng/g, while syn-DP concentrations ranged from ND to 16.30 ng/g with a mean of 8.57 ± 4.55 ng/g (Table 1). The highest DP, anti-DP, and syn-DP concentrations were detected at site T31 (*p* < 0.05), and the lowest DP, anti-DP, and syn-DP concentrations were T26, T27, and T25 (*p* < 0.05) after removing NDs, respectively. The total organic carbon and pH of the soil were also detected, and they were 1.73–4.12% and 6.7–7.8.

Although there has been a dearth of research on DP in soil, DP concentration has been reported in previous research. Mean DP concentrations in soil samples (n = 3) in Harbin City, an urban region in northeastern China, were reported as 0.01 ng/g [45]. DP concentrations were ND to 47.7 ng/g, with a mean of 7.26 ng/g, in surface soils (n = 26) from Qingyuan, South China [33]. DP in soil samples of Jingjin region (n = 7), Shandong Province (n = 27), Hebei Province (n = 29), and Shanxi Province (n = 24) were detected, and mean concentrations were 1.10 ± 2.61, 0.96 ± 2.66, 0.10 ± 0.14, and 0.96 ± 2.66 ng/g, respectively [46]. DP concentration was 0.665–102 ng/g, with a mean of 15.9 ng/g, in surface soil samples (n = 8) of Huai’an City [32]. Compared to previous studies, the mean DP concentration in this study was relatively higher. This is presumably due to the sampling sites being mainly located in a densely populated urban region.

### 3.2. Fractional Abundances of DP Isomers

The FDs of anti-DP (78.79%) were higher than syn-DP (60.61%), and similar findings were reported in other studies [46]. It was not only related to the proportion of syn-DP in DP production, but also differences in properties between syn-DP and anti-DP, such as microbial degradation [47], migration capability [4], physical, and chemical properties [14,48]. The approximated proportion of syn-DP in commercial DP products was reported as 0.20–0.25 [4], and it was also reported as 0.25 and 0.35 in technical products [2,49]. Therefore, the number of syn-DP entering the environment was less than anti-DP, and was more difficult to detect than anti-DP. Previous studies have reported that anti-DP degrades slowly and has lower remobilization potential than syn-DP in soil [50,51]. Compared to syn-DP, anti-DP accumulates readily in surface soil, rather than migrating from the surface to deeper layers, which has been previously reported [29,52]. Conversely, syn-DP was easily degraded by microorganisms compared to anti-DP in soil [2,53]. Thus, the FD of anti-DP was usually higher than syn-DP in soil.

**Figure 2:**
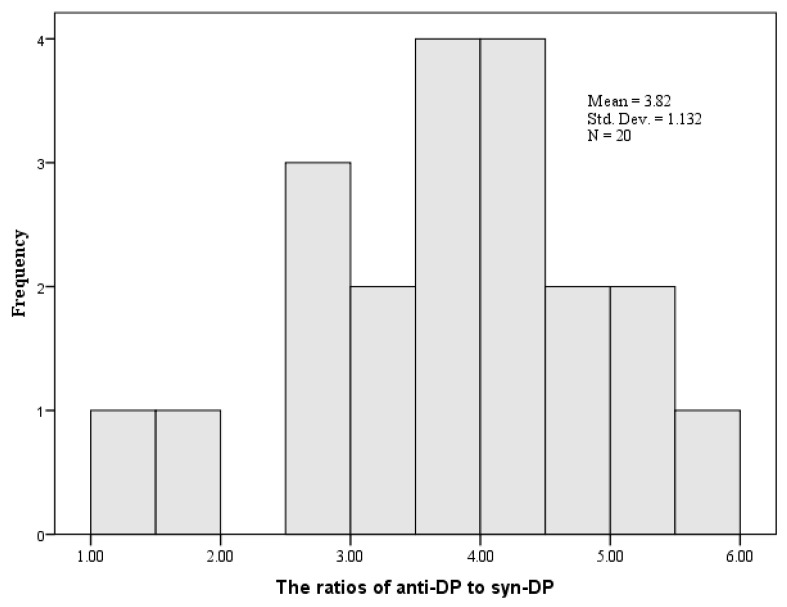
Ratios of anti-DP to syn-DP in the surface soil of Shenyang City.

Anti-DP concentrations (27.01 ± 14.32 ng/g) were consistently greater than syn-DP (8.57 ± 4.55) in soil samples. It was consistent with the previous studies [3,46], relating to the facts that anti-DP occupied most of the proportion in the commercial DP product (about 75–80%), microbial degradation, and migration capability [4,47]. The ratios of anti-DP to syn-DP were 1.50–5.99 and obeyed a normal distribution (Kolmogorov–Smirnov Z = 0.372, Asymp. Sig. (two-tailed) = 0.999) (Figure 2).

The f_syn_ [syn-DP/(anti-DP + syn-DP)] could be used to describe the fractional abundance of DP isomers [4,54], and f_syn_ in soil samples of Shenyang City were shown in Figure S2. f_syn_ values of the soil samples in this study ranged from 0.14 to 0.40, with the mean of 0.22. f_syn_ values varied significantly between soil samples in this study, but it was within the range of previous studies in the different regions of North China (0.11–0.51) [46]. According to previous studies, the approximated f_syn_ in commercial PD products was 0.20–0.25 [4], and the f_syn_ value of technical products from OxyChem was 0.25 [49]. The mean DP values in this study was similar to commercial PD products, suggesting that PD in the soil of Shenyang City was mainly derived from commercial DP products [2]. There was a strong positive correlation between syn-DP and anti-DP in this study (R^2^ = 0.7165) (Figure S3), suggesting that syn-DP and anti-DP were derived from the same sources.

### 3.3. Spatial Distribution of DP

**Figure 3:**
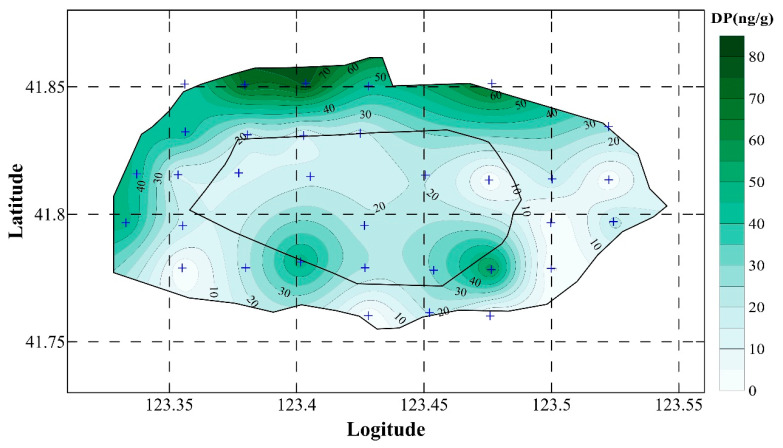
The contour of ∑DP levels (ng/g) in the surface soil in Shenyang City (Kriging interpolation method was applied).

The contour map of DP in the soil in Shenyang City is shown in Figure 3 and used to study the spatial distribution of DP. The contour map showed that DP concentrations were relatively higher in the north and west areas of Shenyang City. This indicates that there were some potential DP pollution sources in the northern or western areas. DP contamination in the ambient environment was not only related to DP use in consumer products, for example, plastic, nylon, and resins [3], but also DP migration. As a “local” pollutant, DP was relatively brutal to migrate over long distances, and DP pollution had a closer relationship with local DP production and use [3]. Although there is no DP production near Shenyang City, DP use in consumer products such as electrical wires and cables, and plastic roofing materials, are likely potential sources. This was consistent with the f_syn_ results in the soil samples of Shenyang City. Meanwhile, with the adjustment to the structure of the industry and urban layout between 2014 and 2018, industries had to almost all relocate from the second-ring road of Shenyang, and some industrial cluster areas had been established in the northern or western region of Shenyang City (outside of the second-ring road). Significant amounts of electrical wires and cables, and plastic roofing materials were used during the construction and production within these regions of the city, resulting in the release of large quantities of DP and accumulation in local soil.

Syn-DP, anti-DP, and DP concentrations in the soil samples from the first- and second-ring roads are shown in Table S2. The results revealed that the mean values of syn-DP (11.42), anti-DP (32.70), and DP (43.76) in the surface soil samples within the second-ring road were all higher (5.90, 18.20, and 22.49) than those within the first-ring road. This means that DP contamination within the second ring road was more severe than that within the first ring road. The mean fsyn value in the second ring road was 0.22, and in the first ring road it was 0.23. Both values were within the proportion of syn-DP in the commercial DP product (0.20–0.25) [4]. This also indicated that the DP in both the first- and second-ring roads mainly came from the raw commercial DP product or commercial DP. Compared with the first ring road, buildings in the second ring road were newer. Many were even under construction. During the construction process, lots of electrical wires, cables, furniture, and plastic materials containing DP were used. Meanwhile, the second-ring road was closer to the industrial zones. The second-ring road area was more vulnerable to the influence of enterprise production. These were the possible reasons why DP pollution was higher in the second-ring road than in the first-ring road. Usually, the spatial distribution and the contaminant level were closely related to land-use areas [38,39,55,56]. The concentration of DP in soil samples of different land-use areas from Shenyang City was investigated (Table S3). DP was also affected by land-use, and mean DP concentrations followed the order traffic > residential > business > park area.

### 3.4. Inventory of DP in Soil

The inventory of DP in the soil was estimated by using the following equation [3]:

(5) $$Inventory=\sum_{i=1}^{n}C_{i}\times \rho_{i}\times (A_{i}/n)\times d_{i}$$

where *n* is the number of land segments in this study; *C_i_* (ng/g) is the mean concentration of DP in the land segment *i*; *ρ_i_* (g dry soil per cm^3^ wet soil) is the soil density of land segment *i*; *A_i_*/*n* is the area of land segment *i*; *d_i_* (cm) is the thickness of sediment *i*. For *ρ_i_*, the values were 1.43 ± 0.077 g dry soil per cm^3^ wet soil [57]. Values of *d_i_* was the sampling depth used in this study (20 cm). The Tyson polygon method by Thissen (1911) [58] was used to delineate the land segment by ArcGIS in this study [59] (Figure S4), and the area of land segment *i* was calculated by ArcGIS 10 (161.32 km^2^). The inventory of DP in surface soil of Shenyang City was calculated according to Equation (5), and the inventory of DP was 1.18 tons. Compared with previously available DP inventories, the DP burden per unit area in surface soil of Shenyang City was higher than those reported in soil of Huai’an and sediment of Lake Ontario [3,53]. This study highlights the need for more research to better understand the extent and magnitude of DP pollution of DP in China and globally.

### 3.5. Human Exposure and Health Risk

Exposure doses and the non-cancer health risk of DP for the five age groups were calculated, and the results are shown in Table S4. Exposure doses for the five age groups ranged from 0.0026 ng/kg-bw/d to 3.44 ng/kg-bw/d, and it followed the order: toddlers > infants > children > teenagers > adults. Compared to dermal absorption, oral intake is the main route of exposure, with exposures that are four orders of magnitude higher than skin absorption. Compared with the previous study of DP exposure [48,60,61], the DP exposure in Shenyang urban surface soil is at a lower level. HQs for the five age groups ranged from 5. 26 × 10^−10^–6.88 × 10^−9^, and they were far less than 1, indicating that the non-carcinogenic risk of DP in urban surface soil of Shenyang is very small or even negligible. Because of the absence of nearby manufacturing facilities and e-waste recycling sites in Shenyang City, combined with the significant contribution of household products to DP exposure, there were less HQs than in the surface soil manufacturing facility area (1.05 × 10^−3^–8.79 × 10^−3^) and e-waste recycling site in China (1.01 × 10^−6^–5.25 × 10^−5^) [40], even significantly lower than indoor dust in China (2.15 × 10^−9^–1.30 × 10^−8^) [41]. The HQs followed the order: toddlers > infants > children > teenagers > adults, and the results suggested that young people are more susceptible to the harmful effects of DP in soil compared with adults, which was consistent with the study of DP exposure via indoor dust [41].

Uncertainty from variations in each parameter (input) is inevitable in health risk assessment [41,60], and the Monte Carlo simulation was used to detect the uncertainty and sensitivity analysis according to the method in some previous studies [41,60]. The concentration of each input to HQ is shown in Figure S5. The results showed that the most uncertain input was the daily ingestion rate of dust (DIR), with a contribution of 47.2%, followed by the DP concentration in the soil (C), which showed a contribution with a range of 27.48–36.56%. Meanwhile, the other input had a lesser contribution (<1.00%). The correlation coefficients between each input and the output were shown in Figure S6, and the results showed that the most influential input was DIR, which ranged from 0.57 to 0.59, followed by the OEF (0.47–0.57) and DP concentration (0.26–0.29), and the Correlation coefficients of other inputs (BW, SAS, AF, BAS) were negligible. So, we should pay more attention on the selection of DIR, and OEF, and more research should be conducted on the selection of health threat and risk assessment parameters.

## 4. Conclusions

The information on the contamination level, distribution, inventory, and health risk of Dechlorane Plus in the urban surface soil of Shenyang City was reported in this study, and it was helpful to better understand the extent and magnitude of the DP pollution of DP in China and globally. Consistent with previous findings, DP was detected at a high prevalence in this study. These results not only reinforce the global ubiquity of DP as an environmental contaminant but also underscore the necessity for continued research to address its implications.

The FD of anti-DP exhibited a higher concentration compared to syn-DP, which can be attributed to a lower proportion in DP production, a greater potential for remobilization, and a higher rate of biodegradation of syn-DP relative to anti-DP. The contour map indicated that DP levels were influenced by urban land use and DP production. These findings indicate that, during the research process of DP, while paying attention to the inherent characteristics of DP, we should also place greater emphasis on the influence of different land use types.

Although the risk is small, we still need to pay attention, especially to children, who are more susceptible to the effects of DP contamination in urban surface soil. Additionally, existing research on health risk assessment parameters for DP remains limited, underscoring the critical need for further toxicological investigations. More toxicological studies on DP are urgently needed. Such studies would not only establish a robust foundation for the precise human health risk evaluations of DP but also enhance the understanding of its ecological implications.

## Figures and Tables

**Table 1:** Syn-DP, anti-DP, and ∑DP concentrations in soil samples (ng/g dry weight).

	Range	Median	Mean	S.D.	FD
syn-DP	ND-16.30	8.56	8.57	4.55	60.61%
anti-DP	ND-61.50	18.71	27.01	14.32	78.79%
∑DP	ND-77.80	24.10	33.60	18.93	78.79%

ND, not detected, FD (%), frequency of detection (%).

## Data Availability

The data that support the findings of this study are available from the corresponding author, upon reasonable request.

## Supplementary Materials

The following supporting information can be downloaded at https://www.mdpi.com/article/10.3390/toxics13050335/s1, Figure S1: The example gas chromatogram of the sample; Figure S2: The fsyn values of all samples; Figure S3: Correlation of syn-DP concentrations plotted against anti-DP concentrations; Figure S4: Land segments delineated using the Tyson polygon method; Figure S5: Analysis of the contribution rate of variance of health risk under different influencing factors of each index; Figure S6: Health risk correlation analysis under different influencing factors of each index; Table S1: Parameters for equations to predicted probability density functions of dust ingestion dose (DEDi) and dust dermal absorption dose (DEDda) of the five age groups; Table S2: Concentrations of syn-DP, anti-DP and ∑DP in soil samples within the first-ring road and second-ring road (ng/g dry weight); Table S3: Concentrations of syn-DP, anti-DP and ∑DP in soil samples of different land-use areas from Shenyang City (ng/g dry weight); Table S4: The exposure dose and non-cancer health risk of DP exposure to dust for five age groups. References [42,62] are cited in Supplementary Materials.
