# Supplementary material for: Contamination Level, Distribution, and Inventory of Dechlorane Plus (DP) in the Surface Soil of Shenyang City, China
Source: Toxics. 2025 Apr 24;13(5):335. doi: 10.3390/toxics13050335 (PMC12115680; doi:10.3390/toxics13050335)
Supplement: Supplementary file 1 [file toxics-13-00335-s001.zip › toxics-3514648-supplementary.pdf]

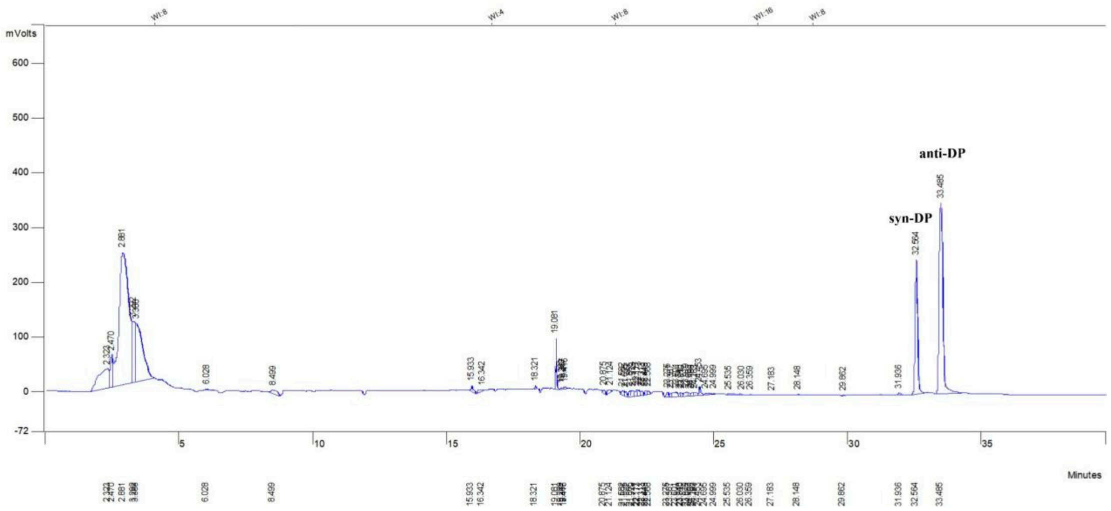

Figure S1. The example gas chromatogram of the sample.

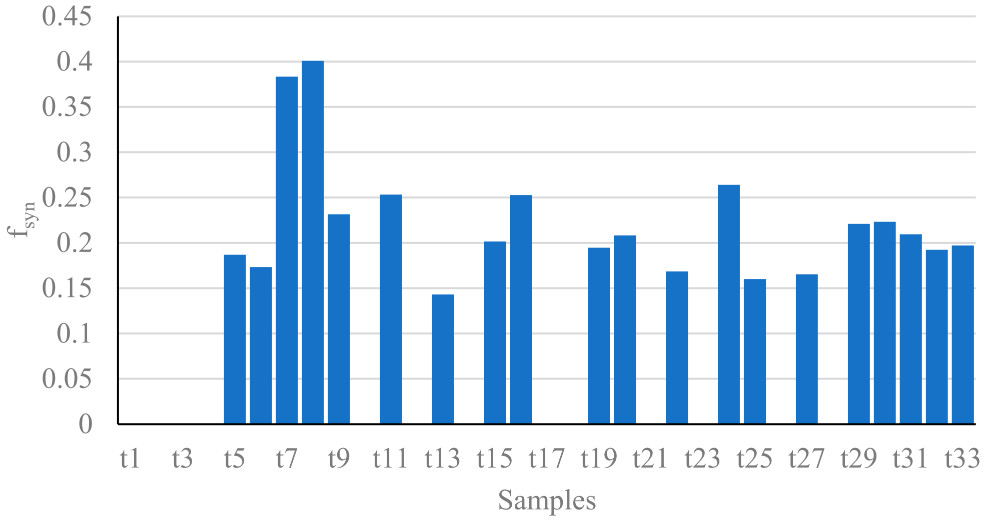

Figure S2. The fsyn values of all samples.

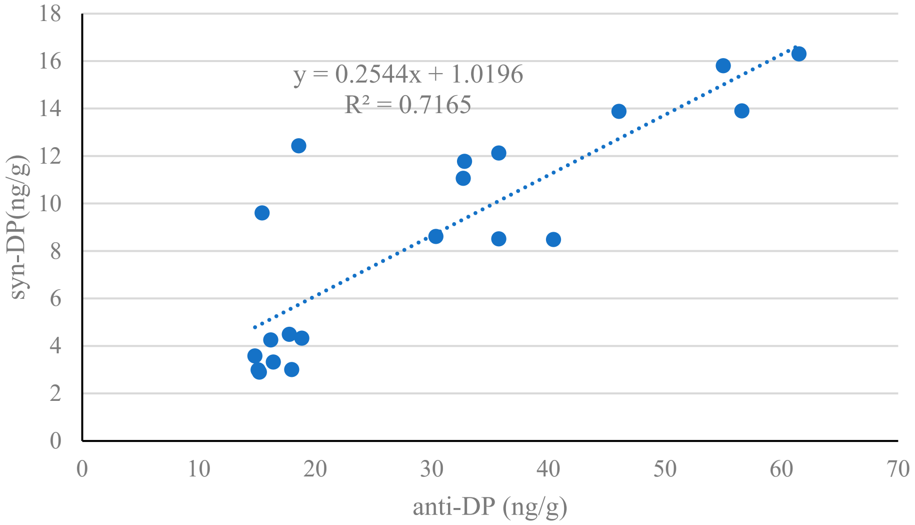

Figure S3. Correlation of syn-DP concentrations plotted against anti-DP concentrations.

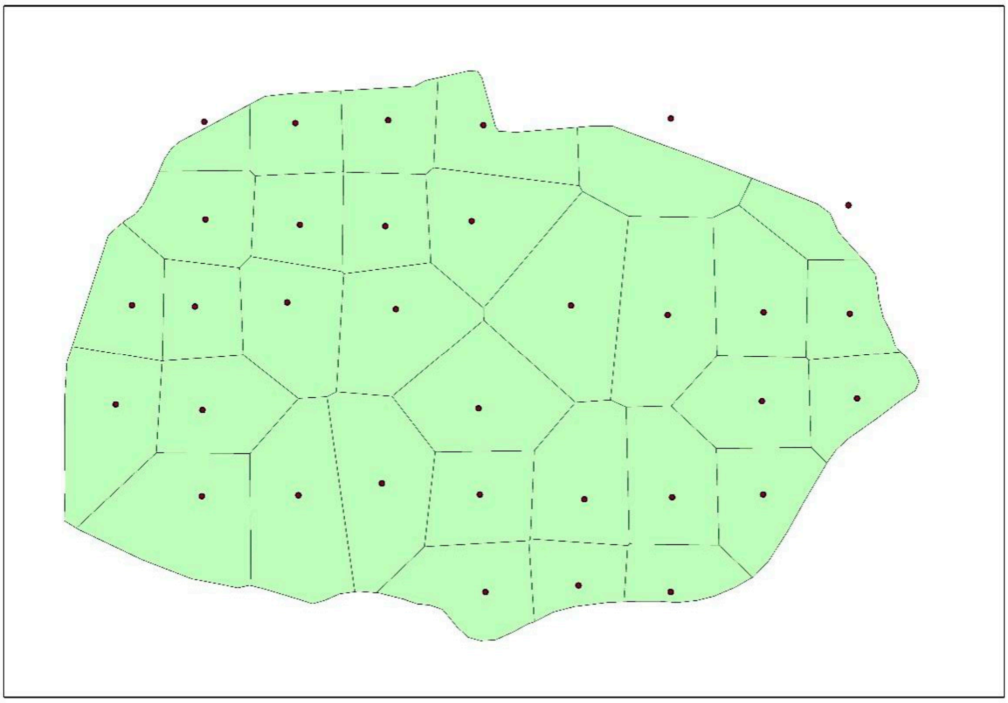

Figure S4. Land segments delineated using the Tyson polygon method.

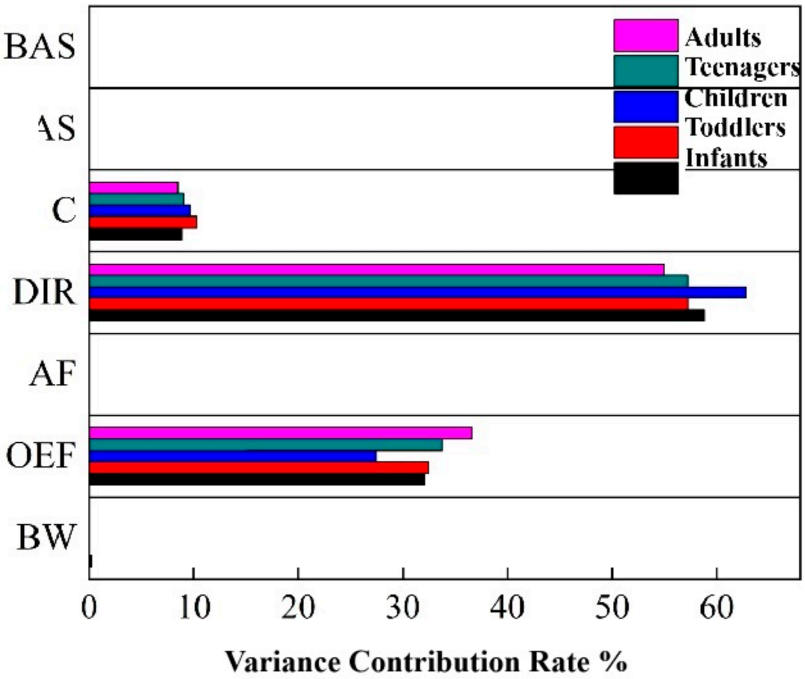

Figure S5. Analysis of the contribution rate of variance of health risk under different influencing factors of each index

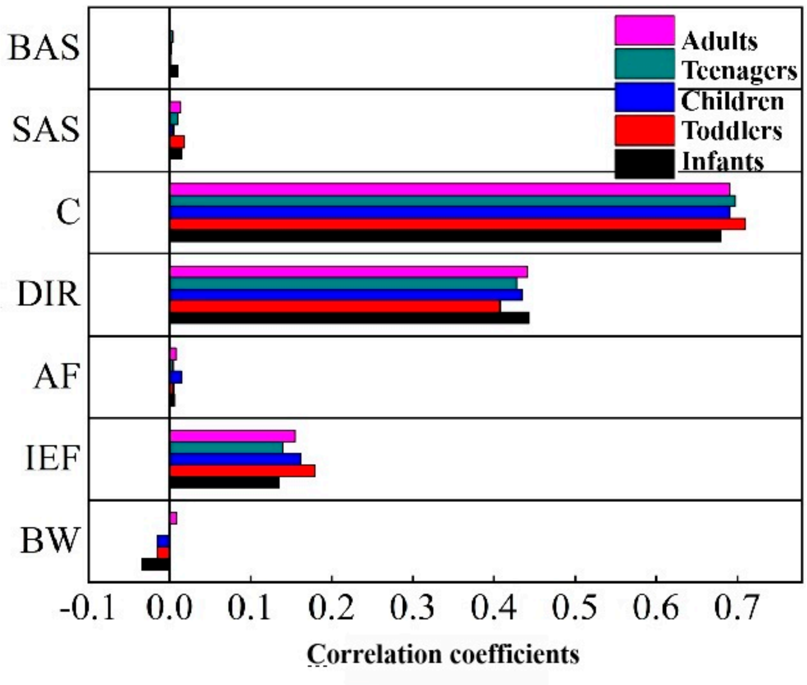

Figure S6. Health risk correlation analysis under different influencing factors of each index

Table S1

Parameters for equations to predicted probability density functions of dust ingestion dose (DEDi) and dust dermal absorption dose (DEDda) of the five age groups.

Category	Distribution	Infants	Toddlers	Children	Teenagers	Adults
Age Range [a,b]		<1	1-5	6-11	12-19	≥20
BW (kg) [a,b]	Lognormal	LN [e] (5,1.01)	LN(16,1.02)	LN(25.2 [f],1.04)	LN(52,1.04)	LN(62.9 [f],1.14)
BSA (cm^2) [b]	Lognormal	LN(801,1.01)	LN(2564,1.02)	LN(3067,1.02)	LN(3692,1.02)	LN(4615,1.03)
SAS (mg cm^{-2} d^{-1}) [b]	Lognormal	LN(0.096,1.01)	LN(0.096,3.41)	LN(0.096,3.41)	LN(0.096,3.41)	LN(0.096,2.67)
AF [c,b]	Lognormal	LN(0.03,1.26)	LN(0.03,1.26)	LN(0.03,1.26)	LN(0.03,1.26)	LN(0.03,1.26)
IEF [b,d]		0.88	0.79	0.79	0.88	0.88
OEF [b,]		0.12	0.21	0.21	0.12	0.12
DIR(mg d^{-1}) [a,b]	Lognormal	LN(0.02,1.09) [f]	LN(0.1,1.6)	LN(0.05,1.6)	LN(0.05,1.6)	LN(0.05,1.6)

a: USEPA. Exposure Factors Handbook: 2011 Edition. Washington, DC, United States Environmental Protection Agency, Office of Research and Development C. 2011. Available from: http://www.epa.gov/ncea/efh/pdfs/efh-complete.pdf.

b: (Johnson-Restrepo and Kannan, 2009)

c: USEPA. Risk Assessment Guidance for Superfund, Human Health Evaluation Manual, Pt E, Supplemental Guidance for Dermal Risk Assessment. vol. 1, USEPA, EPA 540-R-99-005, Washington, DC. Available through: Springfield, VA, National Technical Information Service, PB99-963312. 2004. Available from: http://www.epa.gov/oswer/riskassessment/ragse/index.htm.

d: (Guo and Kannan, 2011)

Table S2. Concentrations of syn-DP, anti-DP and ∑DP in soil samples within the first-ring road and second-ring road (ng/g dry weight).

First-ring road	syn-DP	anti-DP	∑DP	fsyn	Second-ring road	syn-DP	anti-DP	∑DP	fsyn
Range	ND-12.43	ND-40.44	ND-48.92	0.13-0.40	Range	ND-16.30	ND-61.50	ND-77.80	0.17-0.26
Median	3.92	15.44	18.46	0.18	Median	11.42	32.7	43.76	0.22
Mean	5.90	18.20	22.49	0.23	Mean	10.34	33.48	41.75	0.22
S.D.	3.48	7.21	9.53	-	S.D.	4.30	14.78	19.94	-

Table S3. Concentrations of syn-DP, anti-DP and ∑DP in soil samples of different land-use areas from Shenyang City (ng/g dry weight).

		syn-DP（ng/g)	anti-DP（ng/g)	∑DP（ng/g)

Park area	Range	ND-9.60	ND-4044	ND-48.92
	Mean	6.56	22.83	28.29
	S.D.	2.85	10.91	13.42
	FD	0.71	0.86	0.86
Traffic area	Range	ND-15.80	ND-55.00	ND-70.80
	Mean	9.63	34.38	44.01
	S.D.	4.67	15.46	20.13
	FD	0.75	0.75	0.75
Residential area	Range	ND-16.30	ND-61.50	ND-77.80
	Mean	8.96	27.68	34.25
	S.D.	5.01	15.12	20.08
	FD	0.55	0.75	0.75
Business area	Range	ND-11.06	14.41-32.70	14.41-43.76
	Mean	11.06	23.56	29.09
	S.D.	0.00	9.14	14.67
	FD	0.50	1.00	1.00

Table S4 The exposure dose and non-cancer health risk of DP exposure to dust for five age groups.

	Infants	Toddlers	Children	Teenagers	Adults
DED_i（ng/kg/d）	1.32E-02	3.44E-02	1.09E-02	3.20E-03	2.60E-03
DED_{ad}（ng/kg/d）	1.53E-06	2.54E-06	1.90E-06	6.80E-07	7.00E-07
DED_t（ng/kg/d）	1.32E-02	3.44E-02	1.09E-02	3.20E-03	2.60E-03
HQ	2.65E-09	6.88E-09	2.18E-09	6.36E-10	5.26E-10

Reference:

Johnson-Restrepo, B., Kannan, K., 2009. An assessment of sources and pathways of human exposure to polybrominated diphenyl ethers in the United States. Chemosphere 76, 542-548.

Guo, Y., Kannan, K. 2011. Comparative assessment of human exposure to phthalate ethers from house dust in China and the United States. Environmental Science and Technology 45, 3788-3794.
